# A Bilevel Optimization Model Based on Edge Computing for Microgrid

**DOI:** 10.3390/s22207710

**Published:** 2022-10-11

**Authors:** Yi Chen, Kadhim Hayawi, Meikai Fan, Shih Yu Chang, Jie Tang, Ling Yang, Rui Zhao, Zhongqi Mao, Hong Wen

**Affiliations:** 1 College of Electronic Engineering, Chengdu University of Information Technology, Chengdu 610225, China; 2 School of Aeronautics and Astronautics, University of Electronic Science and Technology of China, Chengdu 611731, China; 3 CMA Key Laboratory of Atmospheric Sounding, Chengdu 610225, China; 4 College of Technological Innovation, Zayed University, Abu Dhabi 144534, United Arab Emirates; 5 College of Communication Engineering, Chengdu University of Information Technology, Chengdu 610225, China; 6 Department of Applied Data Science, San Jose State University, San Jose, CA 95192, USA; 7 China Mobile (Chengdu) Industrial Research, Chengdu 610041, China

**Keywords:** edge computing, microgrid, power distribution, cost, optimization

## Abstract

With the continuous progress of renewable energy technology and the large-scale construction of microgrids, the architecture of power systems is becoming increasingly complex and huge. In order to achieve efficient and low-delay data processing and meet the needs of smart grid users, emerging smart energy systems are often deployed at the edge of the power grid, and edge computing modules are integrated into the microgrids system, so as to realize the cost-optimal control decision of the microgrids under the condition of load balancing. Therefore, this paper presents a bilevel optimization control model, which is divided into an upper-level optimal control module and a lower-level optimal control module. The purpose of the two-layer optimization modules is to optimize the cost of the power distribution of microgrids. The function of the upper-level optimal control module is to set decision variables for the lower-level module, while the function of the lower-level module is to find the optimal solution by mathematical methods on the basis of the upper-level and then feed back the optimal solution to the upper-layer. The upper-level and lower-level modules affect system decisions together. Finally, the feasibility of the bilevel optimization model is demonstrated by experiments.

## 1. Introduction

With the vigorous development of renewable energy, the power system structure is becoming increasingly complex and huge, the number of distributed power resources in the distribution network is increasing, and the terminals on the microgrid user side are also various [1,2,3]. The traditional power purchase mode of distribution users in the main network is not adequate for the current power requirements [4,5,6]. In order to adapt to the new situation of continuous development and change, an intelligent microgrid consisting of photovoltaic power generation, a combined cooling heating and power system, an energy storage system and a response load is suggested in the literature [7]. In the microgrid, electrical energy is transmitted in both directions. That is, the electrical energy, according to the actual situation and demand, is able to be effectively transferred to the transmission network; it is not just a one-way transmission [8].

**Figure 1:**
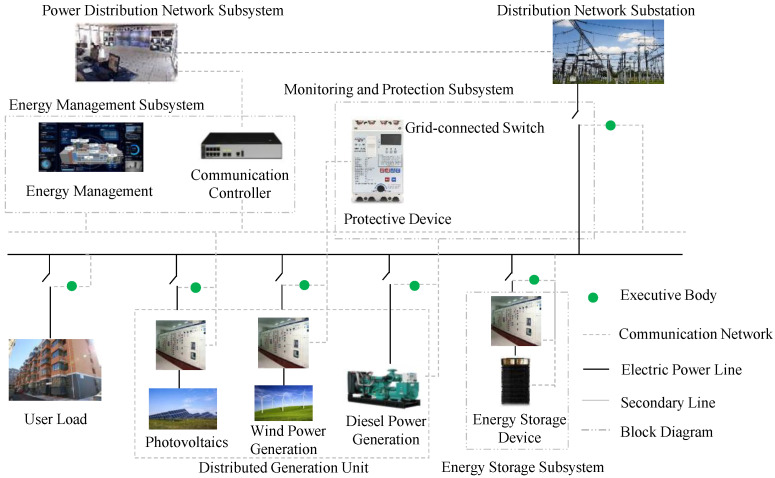
Sketch map of smart microgrid.

A common smart microgrid is an independent system composed of small-scale power generation and distribution systems, as shown in Figure 1, where the distribution system is composed of a distributed generation unit, energy storage, energy converter, related loads, monitoring and protection. The microgrid is usually deployed on the user side, which can avoid voltage instability, blackouts and other trouble [5]. The microgrid usually needs to be connected to the main power grid system through connecting lines. With the addition of various microgrids, the power system architecture has become increasingly complex and huge [6], which not only raises the complexity of equipment deployment and system configuration, but also increases their cost.

Nowadays, with the access to a large number of terminals and the emergence of more users on the demand side, the data flow between electrical equipment terminals and monitoring and control centers, enterprises and power users as well as mobile terminals is growing rapidly [9,10,11,12]. Faced with these new power services and massive data, traditional relational databases have been unable to meet the requirements of efficient data processing. How to improve the efficiency of data processing and power distribution while ensuring the safe and stable operation of the entire power system has been widely valued in the industry [13,14,15,16].

In the deployment process of data processing, computing resources can be deployed simultaneously in the cloud and at the edge of the network, while edge computing is a new computing system and technology that sinks its computing power from the former to the latter to achieve real-time business, efficient data processing, application intelligence, security and privacy protection [17,18,19].

Edge computing has the advantages of a low latency, real-time and efficient data processing capacity [20], data security and privacy protection, personalized configuration and localized processing. It also meets the different application needs of power grid intelligence. Therefore, this paper proposes a bilevel optimization model for microgrid users based on edge computing, which is divided into an upper-level module and a lower-level module. The purpose of the bilevel optimization model is to optimize the power distribution of the microgrid. The decision variables of the microgrid are set in the upper-level module while the optimal solution of the upper-level module is calculated through mathematical methods in the lower-level module. The optimal solution is also fed back to the upper-level module and influences the decision variables. The upper-level and the lower-level affect each other. The combined modules determine the cost-optimal control decision of the microgrid under the load balance condition.

The contributions of this paper are summarized as follows:

1. We put forward a bilevel optimization model, aiming to realize the cost-optimal control decision under the condition of load balancing for microgrid users. The model consists of an upper-level module and a lower-level module.
2. We introduce the modeling process of both the upper-level module and the lower-level module and the model solution procedure in detail. The purpose on the two-layer optimization modules is to optimize the cost of power distribution of microgrids.
3. Extensive simulations are conducted to demonstrate the proposed bilevel optimization model. The results indicate that the proposed model is feasible for the control decision of power distribution of microgrid users.

The remainder of the paper is organized as follows. Section 2 describes the background knowledge of the Dijkstra algorithm. Section 3 illustrates the presented bilevel optimization model in detail. Section 4 introduces the model solution procedure. The experimental results obtained from simulation are given in Section 5. Finally, the paper is concluded in Section 6.

## 2. Background

### 2.1. Dijkstra Algorithm

Inspired by the idea of the greedy algorithm, the Dijkstra algorithm is widely used to obtain the optimal solution of the shortest route problem [21]. The Dijkstra algorithm needs to calculate the shortest distance between all user nodes. The details are as follows.

1. First, the parameters are initialized: start node i=1,2,3…n, destination node j=1,2,3…n, intermediate variable $d_{i,j}=l_{i,j}$, where $d_{i,j}$ represents the intermediate value of the shortest distance solution process and $l_{i,j}$ denotes the distance between adjacent nodes *i* and *j*. If the two nodes are nonadjacent, set $l_{i,j}=+\infty$. Initialize $m_{i,j}=+\infty$, where i≠j and $m_{i,j}$ is the shortest distance from node *i* to node *j*. This shortest distance includes the distances passing through intermediate nodes.
2. Second, compare all distances between adjacent nodes *i* and *j* (i≠j) and let $m_{i,j}=min\left\{d_{i,j}\right\}$, where j=1,2,3…n.
  For all j=1,2,3…n, if $d_{i,j}=m_{i,j}\neq +\infty$, set $N_{i,j}=j$, where $N_{i,j}$ is an intermediate variable. $N_{i,j}=j$ indicates that the node *j* has been compared with node *i*.
  For all j=1,2,3…n, if $N_{i,j}\neq j$, let $d_{i,j}=min\left\{m_{i,k}+l_{k,i},\;d_{i,j}\right\}$, where intermediate node k=1,2,3…n, $m_{i,k}$ is the shortest distance from node *i* to node *k* and $l_{k,i}$ denotes the distance from intermediate node *k* to the adjacent node *i*.
3. Next, judge whether all $N_{i,j}=j$, where j=1,2,3…n. If not, recompare all new distances $l_{i,j}$ except the distance of node $N_{i,j}$. Otherwise, check whether *i* is more than or equal to *n*. If so, the algorithm ends; if not, let i=i+1 and reinitialize the parameters: $d_{i,j}=l_{i,j}$, $m_{i,j}=+\infty$, where j=1,2,3…n and i≠j. Then, continue to execute the algorithm.

## 3. Bilevel Optimization Model

**Figure 2:**
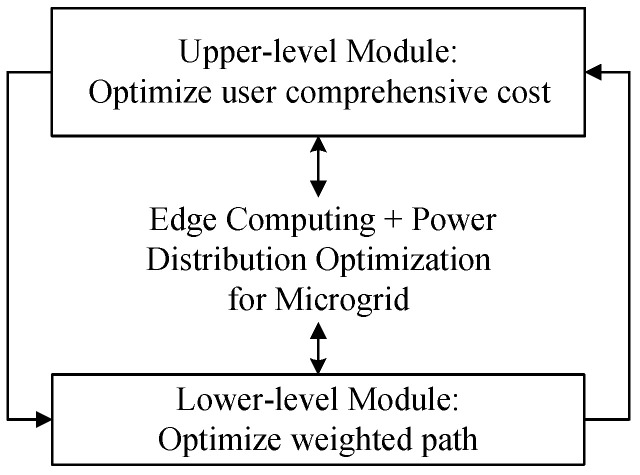
Schematic diagram of bilevel optimization model.

To realize cost-optimal microgrid control decisions under load balance, a bilevel optimization model for a microgrid is put forward in this paper, as shown in Figure 2. The upper-level module mainly establishes the electricity consumption behavior model of microgrid users according to the users’ behavior parameters such as electricity consumption, electricity sale, electricity transmission, electricity consumption time and so on. It considers the comprehensive cost in the process of power grid planning, and then the selection of microgrid nodes is analyzed from the perspective of economic indicators. The lower-level module is mainly to find the optimal solution for the path selection in the network [22,23].

In the bilevel optimization model, the upper-level and lower-level modules are interconnected through intermediate variables, and each module has its own objective function and constraints. The two-layer optimization modules are modeled as follows:

(1) $$\begin{array}{c} \left\{\begin{array}{c} \min\;F=F\left(\alpha_{inv},s\right)\\ s.t\;\;G\left(\alpha_{inv}\right)<=0\\ \;\;\;H\left(\alpha_{inv}\right)=0 \end{array}\right. \end{array}$$

where F(·) is the upper-level objective function, $\alpha_{inv}$ and *s* are the decision variables of the upper-level module, with the caveat that *s* is affected by the lower-level module, and G(·) and H(·) represent the inequalities and constraints of the upper-level module, respectively.

The lower-level module is modeled as follows:

(2) $$\left\{\begin{array}{c} \begin{array}{cc} \min\; & s=f\left(\alpha_{i\mathrm{nv}},\alpha_{nd}\right)\\ s.t\; & g\left(\alpha_{i\mathrm{nv}},\alpha_{nd}\right)<=0\\ \; & h\left(\alpha_{i\mathrm{nv}},\alpha_{nd}\right)=0 \end{array} \end{array}\right.$$

where s(·) represents the lower-level objective function, $\alpha_{inv}$ and $\alpha_{nd}$ are the decision variables of the lower-level module, with the caveat that $\alpha_{inv}$ is affected by the upper-level module, and g(·) and h(·) denote the inequalities and constraints of the lower-level module, respectively.

The overall objective function is shown in Equation (3):

(3) $$\min\;J_{F_{total}}=\lambda_{1}f_{1}+\lambda_{2}f_{2}+\ldots \lambda_{n}f_{n}$$

where $\lambda_{1},\lambda_{2},\cdots ,\lambda_{n}$ are weight ratios from 0 to 1 and $\lambda_{1}+\lambda_{2}+\cdots +\lambda_{n}=1$. The value of $\lambda_{1},\lambda_{2},\cdots ,\lambda_{n}$ is changed by decision-makers according to the emphasis of different objectives.

The purpose of the two-level optimization modules is to optimize the power distribution of the microgrid users. The upper-level module sets the decision variables for the lower-level module. The upper-level module needs to calculate the weight ratio between the attribute values of the microgrid users so that the optimal solution is able to be found in the lower-level optimization module. After the optimal solution is fed back to the upper-level module, it also affects the decision variables simultaneously. The upper-level and lower-level modules influence each other.

### 3.1. Upper-Level Module

The upper-level module is based on the optimization of the comprehensive cost of power distribution at the user node of the microgrid as the objective function, involving parameters of the cost of purchasing electricity, the cost of selling electricity and the cost of electricity transmission. Taking into account the attributes of each user node graph in the microgrid [24] and the economic benefits of the operation of the distribution network [25], the upper-level objective function is represented in Equation (4):

(4) $$\min\;F_{\mathrm{total}}=C_{SA}+C_{ST}+C_{TR}$$

where $F_{total}$ is the comprehensive cost of the distribution network, $C_{SA}$ is the electricity purchasing cost between consumers, $C_{ST}$ is the electricity selling cost and $C_{TR}$ is the electricity transmission cost. The details are as follows.

1. The electricity purchasing cost for consumers is represented in Equation (5):
  (5) $$C_{SA}=\sum_{\tau }^{}\sum_{t}^{}E_{t}^{pr}P_{\tau }^{}$$
  where $E_{t}^{pr}$ is the electricity price at time *t* and $P_{\tau }^{}$ is the exchange power between different microgrids in the τ(τ=1,2,3,4) season.
2. The electricity selling cost is defined in Equation (6):
  (6) $$C_{ST}=\sum_{\tau }^{}\sum_{t}^{}E_{t}^{pr}\left(P_{t}^{pv}+\Delta P_{t}^{pv}\right)$$
  where $E_{t}^{pr}$ is the electricity price at time *t*, $P_{t}^{pv}$ is the photovoltaic power generation at time *t* and $\Delta P_{t}^{pv}$ is the deviation value of photovoltaic power generation at time *t*.
3. The transmission cost is demonstrated in Equation (7):
  (7) $$C_{TR}=\left(\frac{W_{fre}-W_{sre}}{W_{fre}}t\right)\gamma$$
  where $W_{sre}$ is the value of the meter on the input side, $W_{fre}$ is the value of the meter on the output side and γ is the normalization factor.

In addition, the two-level optimization model in the distribution network should also meet the following series of equations and constraints.

1. The line power constraint is defined in Equation (8):
  (8) $$0\leq P_{K}\leq P_{MAX}$$
  where $P_{K}$ is the line power and $P_{MAX}$ is the maximum allowable line power, which is a fixed value determined when the transmission line is constructed.
2. The electric power constraint of the microgrid is shown in Equation (9):
  (9) $$P_{E}=P_{M\_A}+P_{CH}+P_{REG\_self}+P_{CT}$$
  where $P_{E}$ is the electrical power output of the microgrid, $P_{M\_A}$ is the purchased power of the consumers, $P_{CH}$ is the amount of electricity converted by the photovoltaic inverter, $P_{REG\_self}$ is the self-consumption of microgrid distributed renewable energy and $P_{CT}$ is power consumption.
3. The microgrid price constraint is presented in Equation (10):
  (10) $$E_{t}^{pr\_min}<E_{t}^{pr}<E_{t}^{pr\_max}$$
  where $E_{t}^{pr\_min}$ and $E_{t}^{pr\_max}$ are the upper and lower limits of the electricity price, respectively.
4. The microgrid cost constraint is defined in Equation (11).
  (11) $$C_{TR}=\left\{\begin{array}{cc} \frac{\eta_{a0}}{E_{t}^{pr}} & E_{t}^{pr}\in \left(E_{t}^{pr\_min},E_{t}^{pr\_med}\right)\\ \eta_{a1}\times E_{t}^{pr} & E_{t}^{pr}\in \left(E_{t}^{pr\_med},E_{t}^{pr\_max}\right) \end{array}\right.$$
  We see that if the electricity price is less than $E_{prt\_med}$, the electricity price is inversely proportional to the transmission cost, and the higher the electricity price, the lower the transmission cost. If the electricity price is greater than $E_{prt\_med}$, the electricity price is directly proportional to the transmission cost, and the higher the electricity price, the higher the transmission cost.

### 3.2. Lower-Level Module

The main consideration in the upper-level optimal control module was the comprehensive cost. It mainly analyzed the selection of microgrid nodes from the economic indicators. While in the lower-level module, the main goal is to calculate the optimal solution of the path selection through the graph-based path-search algorithm.

1. After the modeling and operation of the upper-level module, we obtain the weight values between the microgrid user nodes. Then, these weight values are combined into a digraph matrix. The lower-level objective function is defined in Equation (12).
  (12) $$\min\;s=\sum_{}^{}l_{ij}$$
  where $l_{ij}$ is an element of the digraph matrix that represents the weight between the microgrid user nodes. $l_{ij}$ is obtained via Equation (3). The digraph matrix is as follows:
  (13) $$\Gamma =\left[\begin{array}{cccc} l_{1,1} & l_{1,2} & \cdots & l_{1,n}\\ l_{2,1} & l_{2,2} & \cdots & l_{2,n}\\ \vdots & \vdots & \vdots & \vdots \\ l_{n,1} & l_{n,2} & \cdots & l_{n,n} \end{array}\right]$$
2. The corresponding lower-level constraints are as follow (14):
  (14) $$\Gamma^{{}^{\prime }}=\left[\begin{array}{cccccc} 0/1 & 0/1 & 0/1 & 0/1 & \cdots & 0/1\\ 0/1 & 0/1 & 0/1 & 0/1 & \cdots & 0/1\\ 0/1 & 0/1 & 0/1 & 0/1 & \cdots & 0/1\\ \vdots & \vdots & \vdots & \vdots & \vdots & \vdots \\ 0/1 & 0/1 & 0/1 & 0/1 & \cdots & 0/1 \end{array}\right]$$
  where “0/1” represents whether the edge of the node of the directed graph exists, which constitutes a constraint graph.

## 4. Model Solution

First, according to the original data of microgrid users, the electricity purchasing cost, electricity selling cost, electricity transmission cost and other parameter values are calculated, and then they are normalized. Second, the optimal solution of the upper model and the weight proportion of each power price parameter is calculated. Next, the optimal solution is obtained through Dijkstra’s algorithm [21,26,27], and finally the optimal distribution scheme is given.

The normalization equation is defined as (15).

(15) $$x=\frac{C_{x}-C_{\min}}{C_{\max}-C_{\min}}$$

where $C_{x}$ is the current cost value (such as $C_{SA}$, $C_{ST}$ and $C_{TR}$), $C_{\max}$ is the maximum of current cost and $C_{\min}$ is the minimum of current cost.

Thus, we obtain the comprehensive cost of the distribution network (i.e., solution of the upper-level objective function):

(16) $$F_{\mathrm{total}\_x}=C_{SA_{-}x}+C_{ST_{-}x}+C_{TR_{-}x}$$

where $C_{SA_{-}x}$, $C_{ST_{-}x}$ and $C_{TR_{-}x}$ are all calculated according to Formula (15).

Then, the weight ratios ($\lambda_{1},\lambda_{2},\cdots ,\lambda_{n}$) are obtained via Equations (17)–(19):

(17) $$\lambda_{C_{SA\_x}}=\frac{C_{SA\_x}}{F_{\mathrm{total}\_x}}$$

(18) $$\lambda_{C_{ST\_x}}=\frac{C_{ST\_x}}{F_{\mathrm{total}\_x}}$$

(19) $$\lambda_{C_{TR\_x}}=\frac{C_{TR\_x}}{F_{\mathrm{total}\_x}}$$

If we set the start node as *A* and select the adjacent node as *B* on any branch of the destination node, the weighted objective function value $J_{F_{\mathrm{total}\;\_x}}$ (i.e., the overall objective function) from *A* to *B* is obtained via Equation (20):

(20) $$\begin{array}{c} J_{F_{\mathrm{total}\_x}}=C_{A\cdot \mathrm{SA}\_x}\times \lambda_{C_{SA\_x}}+C_{B\cdot ST\_x}\times \lambda_{C_{ST\_x}}+C_{AB\cdot TR\_x}\times \lambda_{C_{TR\_x}} \end{array}$$

where $C_{A\cdot \mathrm{SA}\_x}$ is the electricity purchasing cost of node *A*, $C_{B\cdot ST\_x}$ is the electricity selling cost of node *B* and $C_{AB\cdot TR\_x}$ is the electricity transmission cost between node *A* and node *B*.

After we calculate the weighted objective function value $J_{F_{\mathrm{total}}}$ between all adjacent nodes, we obtain the digraph matrix Γ.

Last, the best solution is found through Dijkstra’s algorithm, introduced in Section 2.1.

## 5. Simulated Results

To verify the feasibility of the bilevel optimization model for the microgrid proposed in this paper, we designed the following simulation experiment.

Taking the monthly electricity consumption data as the experimental dataset, where Table 1 shows some of the original monthly power consumption data of user nodes, we conducted some simulations.

**Figure 3:**
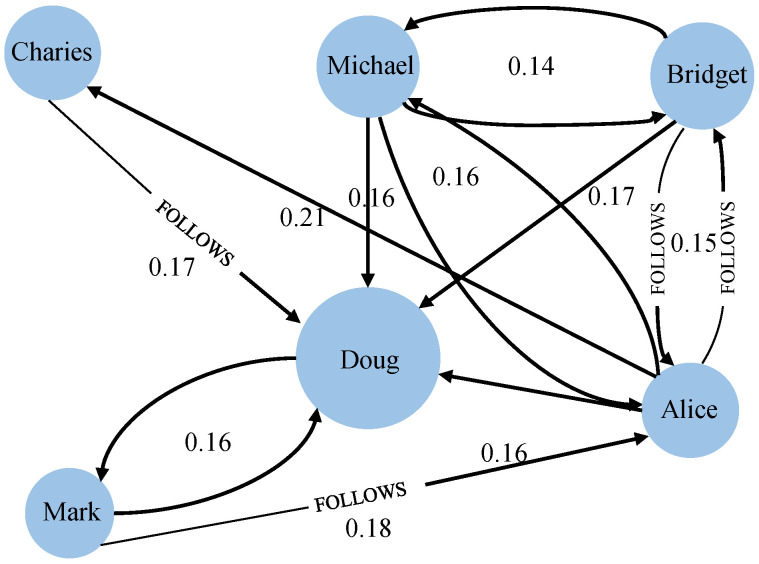
A relationship diagram of microgrid users. The direction of the arrow indicates the transmission direction of electricity. For example, “Charies ⟶ Doug” denotes that Charies can transmit electricity to Doug but Doug cannot transmit electricity to Charies, because there is no arrow going from Doug to Charies. “Mark ⇆ Doug” indicates that they can transmit electricity to each other because there are two-way arrows between them.

**Figure 4:**
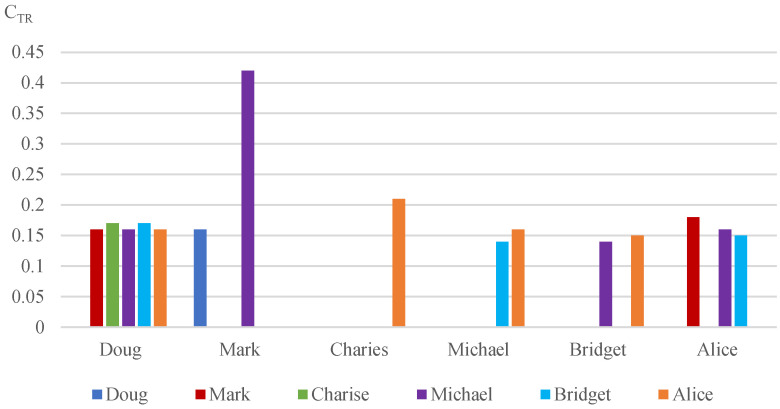
Users’ electricity transmission cost $C_{TR}$.

We selected six users’ node (customer) information to do the simulation. Figure 3 is a relationship diagram of the six microgrid users. The original dataset was processed with the precedence diagram method [28]. The mean-shift clustering algorithm [29] was employed to extract data features of users so that the electricity purchasing price was in the range between 0.28 (CNY/kWh) and 0.84 (CNY/kWh) at time *t*, the electricity selling price was in the range between 0.13 (CNY/kWh) and 0.66 (CNY/kWh), the photovoltaic power generation was 260 W and the line loss rate was in the range between 3% and 8%. Figure 4 displays one group of data of the microgrid users’ electricity transmission cost.

**Figure 5:**
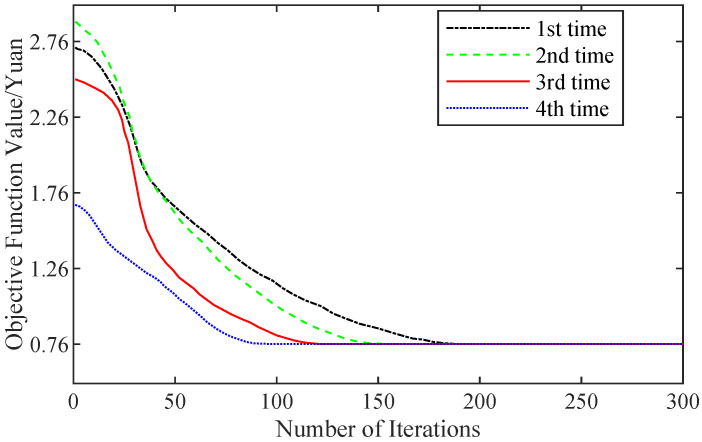
Convergence of objective function versus iterations.

We utilized the particle swarm algorithm [27] to find the optimal solution, where the learning factor was set to 2, the inertia weight was set to 0.5 and the maximum number of iterations was set to 300. We conducted four experiments, and the convergence curves of the objective function are demonstrated in Figure 5. We see that after repeating the operation four times, the values of the objective function all converge to 0.76, so this output is the optimal solution. The parameters corresponding to the optimal solution were $C_{SA}=0.28$, $C_{ST}=0.31$ and $C_{TR}=0.17$.

Then, according to the weight ratio Equations (17)–(19), we obtained the optimal weight ratios as $\lambda_{C_{SA\_X}}=0.368,\lambda_{C_{ST\_X}}=0.407$ and $\lambda_{C_{TR\_X}}=0.223$. The weight ratios corresponding to different $C_{SA}$, $C_{ST}$ and $C_{TR}$ are demonstrated in Table 2.

In addition, we calculate the weighted objective function value of microgrid users with Equation (20), as shown in Table 3.

Where “inf” means that there is no direct connection between the two nodes, i.e., the two nodes are in an unreachable state. In this case, the weight ratio between the parameters is 1:1:1.

**Figure 6:**
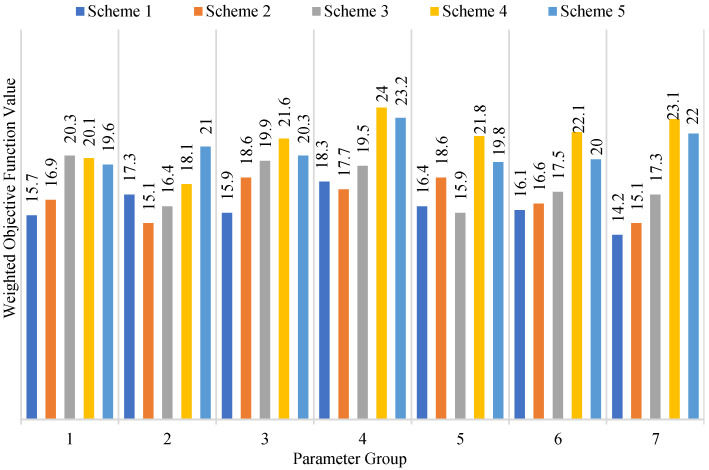
Comparison of weighted comprehensive costs with different schemes.

If setting Bridget as the start node and Doug as the destination node, from Table 3 and Figure 3, we find that there are five paths from Bridget to Doug, i.e., five schemes in total as presented in Table 4. Then, from Table 3 and Table 4, we get the total weighted objective function value on the five paths, as shown in Figure 6.

From Figure 6, we see that the weighted comprehensive cost of scheme 1 corresponding to parameter group 7 is minimal. Thus, it is the optimal solution from Bridget to Doug, that is, when $C_{SA}=0.28,C_{ST}=0.31$ and $C_{TR}=0.17$, the comprehensive cost is optimal from Equation (4). In addition, from Figure 6, for the scheme recommendations with fixed parameters, scheme 2 is the optimal option for the second group of parameters, and scheme 3 is the optimal option for the fifth group of parameters.

## 6. Conclusions

To realize the cost-optimal control decision of microgrids under the condition of load balance, this paper proposed a bilevel optimization model for microgrid users based on edge computing. The modeling process of both the upper-level module and the lower-level module was introduced in detail. The model solution was also provided. Finally, the experimental results indicated that the presented bilevel optimization model was feasible for the control decision of power distribution of microgrid users.

In the follow-up work, we will further optimize the bilevel model, for instance, considering more complex microgrid structures, more user nodes and more parameters. In addition, we think that it is meaningful to analyze the impact of different attributes of each user on the performance of the bilevel model.

## Figures and Tables

**Table 1:** Some of the original monthly power consumption (kWh) data of microgrid users.

Customer	Jan.	Feb.	Mar.	Apr.	May	Jun.	Jul.	Aug.	Sep.	Oct.	Nov.	Dec.	Jan.	Feb.	Mar.	Apr.	May
675	434	441	438	412	402	591	584	583	582	584	593	408	438	445	492	814	308
676	435	434	419	414	410	590	595	590	591	400	403	423	444	442	488	484	304
677	434	445	434	424	404	534	551	538	581	534	580	534	538	549	530	535	591
678	433	429	419	593	585	544	531	543	558	555	559	541	541	534	541	544	545
679	438	403	599	590	531	532	549	553	545	535	584	408	434	449	499	483	498
680	439	444	443	430	433	404	402	588	590	595	403	444	440	488	498	341	353
681	480	499	443	434	438	415	409	419	421	425	413	439	454	444	484	310	303
682	481	453	449	429	403	404	594	402	598	403	418	434	450	448	495	485	485
683	482	485	443	450	442	434	423	413	411	413	422	445	435	485	499	498	309
684	483	435	481	445	443	423	419	419	401	594	589	593	534	532	543	534	582
685	400	452	483	419	418	403	585	581	583	533	544	533	542	583	550	549	558
686	485	424	412	598	585	593	583	544	543	583	401	432	448	490	308	814	341
687	484	444	444	441	422	422	409	409	413	405	411	448	435	305	328	313	322
688	483	443	435	430	438	413	415	413	403	424	429	443	459	300	333	329	320
689	488	449	433	433	455	444	432	433	443	439	453	441	484	494	328	309	312

**Table 2:** Weight ratios of different cost.

		Cost Parameter	$C_{SA}$	$C_{ST}$	$C_{TR}$
	Weight Ratios				
Parameter Group					
1			0.263	0.625	0.425
2			0.163	0.431	0.235
3			0.218	0.342	0.117
4			0.284	0.523	0.425
5			0.412	0.321	0.241
6			0.251	0.325	0.415
7			0.368	0.407	0.223

**Table 3:** The weighted objective function value between user nodes in the microgrid.

		Destination Nodes	Doug	Mark	Charies	Michael	Bridget	Alice
	Weighted Objective Function Value							
Initial Nodes								
Doug			0	6.2	inf	inf	inf	inf
Mark			6.2	0	inf	inf	inf	19.6
Charies			4.3	inf	0	inf	inf	inf
Michael			4.6	inf	inf	0	9.6	6.8
Bridget			15.1	inf	inf	9.6	0	10.6
Alice			6.7	9.5	26.2	6.8	10.6	0

**Table 4:** Different schemes from start node to destination node.

Scheme	Path
1	Bridget → Michael → Doug
2	Bridget → Doug
3	Bridget → Alice → Doug
4	Bridget → Michael → Alice → Doug
5	Bridget → Alice → Michael → Doug

## Data Availability

The data used to support the findings of this study are included within the article.
